# Orai1 and Orai3 Mediate Store-Operated Calcium Entry Contributing to Neuronal Excitability in Dorsal Root Ganglion Neurons

**DOI:** 10.3389/fncel.2017.00400

**Published:** 2017-12-19

**Authors:** Dongyu Wei, Yixiao Mei, Jingsheng Xia, Huijuan Hu

**Affiliations:** Department of Pharmacology and Physiology, Drexel University College of Medicine, Philadelphia, PA, United States

**Keywords:** store-operated calcium channels, dorsal root ganglia, Orai1, Orai3, neuronal excitability, pain

## Abstract

Store-operated calcium channels (SOCs) are highly calcium-selective channels that mediate calcium entry in various cell types. We have previously reported that intraplantar injection of YM-58483 (a SOC inhibitor) attenuates chronic pain. A previous study has reported that the function of SOCs in dorsal root ganglia (DRG) is enhanced after nerve injury, suggesting that SOCs may play a peripheral role in chronic pain. However, the expression, functional distribution and significance of the SOC family in DRG neurons remain elusive and the key components that mediate SOC entry (SOCE) are still controversial. Here, we demonstrated that the SOC family (STIM1, STIM2, Orai1, Orai2, and Orai3) was expressed in DRGs and STIM1 was mainly present in small- and medium-sized DRG neurons. Using confocal live cell imaging, Ca^2+^ imaging and electrophysiology techniques, we demonstrated that depletion of the endoplasmic reticulum Ca^2+^ stores induced STIM1 and STIM2 translocation, and that inhibition of STIM1 or blockage of Orai channels with pharmacological tools attenuated SOCE and SOC currents. Using the small inhibitory RNA knockdown approach, we identified STIM1, STIM2, Orai1, and Orai3 as the key components of SOCs mediating SOCE in DRG neurons. Importantly, activation of SOCs by thapsigargin induced plasma membrane depolarization and increased neuronal excitability, which were completely abolished by inhibition of SOCs or double knockdown of Orai1 and Orai3. Our findings suggest that SOCs exert an excitatory action in DRG neurons and provide a potential peripheral mechanism for modulation of pain hypersensitivity by SOC inhibition.

## Introduction

Intracellular Ca^2+^ is crucial in regulating numerous neuronal functions, including neuronal development, differentiation, excitation, neurotransmitter release, and neuronal cell death (Ghosh and Greenberg, 1995; Berridge, 1998, 2012). An increase in intracellular Ca^2+^ concentration is elicited through intracellular Ca^2+^ release and Ca^2+^ influx through Ca^2+^-permeable channels located in the plasma membrane. Changes of intracellular Ca^2+^ concentration are associated with neurological disorders including Alzheimer’s disease, Parkinson’s disease, stroke and pain (Wojda et al., 2008; Hagenston and Simonetti, 2014; Lai et al., 2014; Desmet et al., 2017). It has been reported that peripheral nerve injury impairs the cytoplasmic Ca^2+^ homeostasis in sensory neurons (Fuchs et al., 2005, 2007; Pan et al., 2016) and elevated cytosolic calcium has been linked to inflammatory chronic pain and diabetic neuropathy (Kostyuk et al., 2001; Huang et al., 2002; Lu and Gold, 2008). These studies suggest that disordered Ca^2+^ homeostasis is associated with pain hypersensitivity. Sensory neurons express a variety of voltage-gated Ca^2+^ channels (VGCC) and ligand-gated Ca^2+^ channels (Lirk et al., 2008; Park and Luo, 2010). However, recent studies have shown that SOCs also mediate Ca^2+^ influx in sensory neurons (Rigaud et al., 2009; Gemes et al., 2011).

Store operated calcium channels are highly Ca^2+^ selective cation channels that can be activated by depletion of Ca^2+^ stores from ER (Putney, 2010). SOCs are composed of two Ca^2+^ sensors, stromal interaction molecules (STIM)1 and STIM2, located on the surface of ER membrane, and three pore-forming subunits Orai1/2/3, known as Ca^2+^ release-activated Ca^2+^ channels (CRAC channels), located in the plasma membrane (Lewis, 2007). We and others have demonstrated that systemic administration, intrathecal or intraplantar injection of YM-58483, a potent SOC inhibitor, attenuates chronic pain (Gao et al., 2013; Qi et al., 2016). Previous studies including ours have shown that SOCs are functional in DRG neurons (Gao et al., 2013) and the function of SOCs in sensory neurons is enhanced after nerve injury (Gemes et al., 2011). These findings suggest that SOCs play both central and peripheral roles in chronic pain. Although characteristics of SOCs have been established in spinal cord neurons and astrocytes (Xia et al., 2014; Gao et al., 2016), the molecular components of SOCs that mediate SOC entry (SOCE) in dorsal root ganglion (DRG) neurons remain elusive. While a previous study has suggested that Orai1 may be involved in SOCE in DRG neurons (Gemes et al., 2011), a recent report has indicated that TRPC3 also contributes to SOCE in DRG neurons (Alkhani et al., 2014). Moreover, the functional significance of SOC activation is still unclear.

Here, we have demonstrated that the SOC family is expressed in DRGs, and have found that SOCs are functional mainly in nociceptors. We have identified STIM1, STIM2, Orai1, and Orai3 as the key components of SOCs mediating SOCE in DRG neurons. Furthermore, our results have indicated that activation of SOCs induces membrane depolarization and increases neuronal excitability of DRG neurons, which are completely abolished by double knockdown of Orai1 and Oria3. Together, our findings have revealed an excitatory role of SOCs in DRG neurons and suggest that SOCs may be involved in the pain process.

## Materials and Methods

### Animals

All experiments were performed in accordance with the guidelines of the National Institutes of Health, the Committee for Research and Ethical Issues of IASP, and were approved by the Animal Care and Use Committee of Drexel University College of Medicine. Pregnant CD1 and C57BL/6 mice were purchased from Charles River (Wilmington, MA, United States) and individually housed in standard cages on a 12 h light/dark cycle. Neonatal and adult mice from both CD1 and C57BL/6 mice were used for cell cultures (there was no difference in SOCE between these two strains). C57BL/6 adult mice were used for immunostaining and Western blot experiments.

### Cell Culture

Primary cultures of DRG neurons were prepared from neonatal [postnatal day 1 (P1) or P2] mice and adult mice (6–8 weeks of age) (Gao et al., 2013). Briefly, mice were decapitated after the induction of anesthesia. A laminectomy was performed and DRGs were collected in cold (4°C) Hanks balance salt solution (HBSS; Corning cellgro, Manassas, VA, United States) (in mM: 137 NaCl, 5.4 KCl, 0.4 KH_2_PO_4_, 1 CaCl_2_, 0.5 MgCl_2_, 0.4 MgSO_4_, 4.2 NaHCO_3_, 0.3 Na_2_HPO_4_, and 5.6 glucose) containing 10 mM HEPES (Sigma–Aldrich, St. Louis, MO, United States). Ganglia were incubated for 30 min at 37°C in HBSS containing 15 U/ml papain (Worthington Biochemical, Lakewood, NJ, United States) and 0.5 mg/ml collagenase (Sigma–Aldrich). Ganglia were then rinsed three times with HBSS, and placed in culture Neurobasal A (Invitrogen) medium containing 2% heat-inactivated horse serum (Invitrogen), 0.2 mM L-glutamax-1 (Invitrogen) and 2% B-27 (Invitrogen). Ganglia were mechanically dissociated by gently triturating with a pipette and the cell suspension was filtered with a 40 μm cell strainer. The resulting DRG neurons were plated onto poly-D-lysine- and laminin-coated coverslips or plates. Cells were maintained at 37°C in a humidified atmosphere containing 5% CO_2_ for 16–72 h.

### Transfection

For knocking down STIM1/2 and Orai1/2/3, acutely isolated DRG neurons were electroporated using a mouse neuron nucleofector kit according to the manufacturer’s instructions (Lonza Group, Basel, Switzerland) as described in our previous study (Xia et al., 2014). Briefly, neurons were transfected with 12 μg per 3 × 10^6^ cells of siRNA targeting STIM1, STIM2, Orai1, Orai2, Orai3, or scramble siRNA (all from Life technologies). For some Ca^2+^ imaging experiments, neurons were co-transfected with GFP plasmid and targeting siRNA or scramble siRNA. Ca^2+^ imaging and Western blot analysis were performed 48–72 h after transfection. For transfection of STIM1-YFP or STIM2-YFP (generous gifts from Dr. Gill, Temple University, PA, United States), the DRG neurons were transfected with 2 μg STIM1-YFP or STIM2-YFP per 3 × 10^6^ cells and were seeded on 15 mm glass coverslips. 16 h after transfection, the medium was removed and neurons were fed with fresh culture medium.

### Real-Time PCR Analysis of mRNA Expression

Real-time PCR was performed as described in our previous study (Xia et al., 2014). Total RNA was extracted from DRGs or cultured DRG neurons using TRIzol Reagent (Molecular Research Center, Cincinnati, OH, United States). The RNA concentration was determined by optical density at 260 nm. Total RNA was reverse transcribed into cDNA for each sample using a Fermentas cDNA synthesis kit (Thermo Scientific, Rockford, IL, United States) following the manufacturer’s instructions. Specific primers for mouse STIM1 (Mm00774349_m1), STIM2 (Mm01223103_m1), Orai1 (Mm00774349_m1), Orai2 (Mm04214089_s1), Orai3 (Mm01612888_m1), and GAPDH were purchased from Applied Biosystems (Foster City, CA, United States). Real-time quantitative PCR (RT-qPCR) was performed in a 7900HT fast real-time PCR system (Applied Biosystems) under the following conditions: 5 min of initial denaturation at 96°C, then 35 cycles of 96°C for 30 s, 55°C for 30 s, and 72°C for 1.5 min. The threshold cycle for each gene was determined and analyzed using the relative quantitation software (Applied Biosystems). The relative expression of the target genes was calculated using the 2 (–Delta Delta C_T_, 2^-ΔΔC_T_^) method. The mRNA levels of STIM1, STIM2, Orai1, Orai2, and Orai3 were normalized to the housekeeping gene GAPDH.

### Western Blot Analysis

Mouse DRGs were collected and homogenized using a Dounce homogenizer in an ice-cold radio immunoprecipitation assay (RIPA) buffer containing 50 mM Tris HCl, 150 mM NaCl, 0.2 mM EDTA, 1% Triton X-100, 2% sodium dodecyl sulfate, 1% deoxycholate, 0.1 mM phenylmethanesulfonyl fluoride and protease inhibitor cocktails (Thermo Fisher Scientific, Waltham, MA, United States). For cultures, DRG neurons were washed in PBS and lysed in RIPA buffer. The lysed tissues and neurons were sonicated at a constant intensity of 2.5 for 10 s, and centrifuged at 12,000 × *g* (4°C) for 5 min. Total protein concentrations were determined using a Pierce bicinchoninic acid protein assay kit (Thermo Fisher Scientific) following the manufacturer’s instructions. Protein samples were heated at 95°C for 10 min, electrophoresed in 10% SDS polyacrylamide gel, and transferred onto Nitrocellulose membranes (Bio-Rad, Hercules, CA, United States). Blots were blocked with Odyssey blocking buffer (TBS) for 1 h at room temperature and probed with rabbit anti-STIM1 (1:4000, Cell Signaling, Danvers, MA, United States), anti-STIM2 (1:4000, ProSci, Poway, CA, United States), anti-Orai1 (1:1000, ProSci), anti-Orai2 (1:1000, Prosci), anti-Orai3 (1:500, ProSci) and anti-beta actin (1:20,000, Thermo Fisher Scientific) primary antibodies at 4°C overnight. The blots were washed and incubated for 1 h at room temperature with IRDye Donkey anti-Rabbit/Mouse secondary antibodies (1:10,000, LI-COR). The bands were quantified using Odyssey Image Studio Software (LI-COR, Inc., Lincoln, NE, United States).

### Immunofluorescent Staining

CD1 or C57BL/6 mice were deeply anesthetized with ketamine and perfused transcardially with saline followed by 4% paraformaldehyde in 0.1 M phosphate buffer (PB) solution (pH 7.4). The L4 and L5 DRGs were extracted, post-fixed in 4% paraformaldehyde PB solution at 4°C overnight, and then moved to 30% sucrose PB solution at 4°C until immersion. The DRGs were frozen in Tissue-Tek O. C. T. compound (Sakura Finetek, VWR, Radnor, PA, United States) on dry ice, and cut into 25 μm thick slices. Sections were blocked with PBS containing 5% normal goat serum (NGS) and 0.3% Triton-X 100 (blocking solution) for 1 h, and were then incubated with the following primary antibodies (all diluted in the blocking solution) at 4°C overnight: Cy3-conjugated NeuN (1:100, EMD Millipore, Billerica, MA, United States), STIM1 (1:100, rabbit, Cell Signaling), NF200 (1:1000, mouse, Sigma–Aldrich), CGRP (1:200, goat, Abcam, Cambridge, United Kingdom) and IB4 (3 μg/ml, Sigma–Aldrich). After three washes in PBS, the sections were incubated with secondary antibodies (Alexa Fluor, ThermoFisher, Waltham, MA, United States) at room temperature for 1 h in the blocking solution. DRG sections were mounted onto glass slides after washes and coverslips were applied using mounting media (SouthernBiotech, Birmingham, AL, United States) after the slices were air dry. Images were captured using the Olympus FLUOVIEW FV1000 confocal microscope equipped with a 30× oil-immersion objective.

### Ca^2+^ Imaging

Ca^2+^ imaging was performed as we previously described (Xia et al., 2014). Briefly, DRG neurons were loaded with 4 μM fura-2AM (Life Technologies) for 30 min at room temperature in HBSS, washed and further incubated in a bath solution containing (in mM) 140 choline-Cl, 10 KCl, 2 CaCl_2_, 1 MgCl_2_, 10 HEPES, and 10 glucose (pH 7.4) for 20 min. Coverslips were mounted in a small laminar-flow perfusion chamber (Model RC-25, Warner Instruments, Hamden, CT, United States) and continuously perfused at 6–7 ml/min with the bath solution. Images were acquired at 3-s intervals at room temperature (20–22°C) using the software MetaFluor 7.7.9 (Molecular Devices, Sunnyvale, CA, United States). The fluorescence ratio was determined as the fluorescence intensities excited at 340 and 380 nm with background subtraction. Only one recording was made from each coverslip. The free Ca^2+^ concentration was calculated by the formula [Ca^2+^] = *K*_d_^∗^β^∗^(*R*–*R*_min_)/(*R*_max_–*R*), where β = (*I*_380max_)/(*I*_380min_). *R*_min_, *R*_max_, and β were determined by *in situ* calibration, as described previously (Fuchs et al., 2005), and 224 was used as the dissociation constant *K*_d_ (Grynkiewicz et al., 1985).

### Live Cell Confocal Imaging

Time-lapse imaging was performed in STIM1-YFP or STIM2-YFP transfected DRG neurons. All fluorescence images were captured 24–48 h post transfection using the Olympus FLUOVIEW FV1000 confocal microscope equipped with a 60× oil-immersion objective. Images were acquired at 60-s intervals using a 515-nm laser line for YFP excitation, and YFP emission through a 535- to 565-nm window. Regions of interest were randomly selected by drawing a line along the plasma membrane to measure the intensity, and the same line was moved to the cytosol area to measure the intensity changes before and after treatment of thapsigargin (TG). The translocation of STIM1-YFP and STIM2-YFP was quantified using ImageJ. Values were normalized to intensity at time 0 of the same neuron.

### Electrophysiological Recording

Standard whole-cell recordings were performed with an EPC 10 amplifier and PatchMaster software (HEKA Elektronik, Lambrecht, Germany) at room temperature as described previously (Hu and Gereau, 2003). For recording SOC currents, a gap-free protocol was used, in which the membrane voltage was held at -70 mV without depolarization or hyperpolarization voltage pulses applied. The electrode solution contained (in mM) 125 CsMeSO_4_, 8 MgCl_2_, 10 BAPTA, 10 HEPES, 3 Na_2_ATP, 0.3 Na_2_GTP, and 0.002 TG, pH 7.4; the bath solution was Tyrode’s solution containing (in mM) 140 NaCl, 5 KCl, 2 CaCl_2_, 1 MgCl_2_, 10 Hepes, and 5.6 glucose (Xia et al., 2014). A divalent-free (DVF) bath solution was also used for recording SOC currents, which was prepared by removing CaCl_2_ and MgCl_2_ from the Tyrode’s solution and adding 0.1 mM EGTA (Gemes et al., 2011). For the current-voltage (I–V) curve recordings, a Na^+^/K^+^/Ca^2+^ free solution was used to eliminate cation influx through voltage-gated cation channels and prepared by replacing NaCl, KCl, and CaCl_2_ with 145 NMDG and 0.1 EGTA. Currents were recorded using a voltage ramp protocol from –100 to 80 mV for 100 ms. All recorded neurons were held at 0 mV. For current-clamp recordings, the electrode solution contained (in mM) 140 KMeSO_3_, 2 MgCl_2_, 2 BAPTA, 10 HEPES, 3 Na_2_ATP, 0.3 Na_2_GTP, pH 7.4. The bath solution was also Tyrode’s solution. Action potentials were generated by current injection from a holding potential of –65 mV. The holding potential was maintained by current injection throughout the entire recording process. Intrinsic excitability was measured every 10 s using a constant amplitude small depolarizing pulse. The amplitude that evoked two to five action potentials during the pre-drug period was selected and remained constant throughout the recording. The spike frequency was measured by counting the number of spikes within a 1-s depolarizing pulse (Hu et al., 2006). Electrode resistances were 3–5 MΩ, and most neurons had series resistance from 4 to 15 MΩ. Only one DRG neuron was recorded in each coverslip.

### Drug Application

Thapsigargin, gadolinium chloride (GdCl_3_), 2-APB, capsaicin, menthol, and AITC were purchased from Sigma (St. Louis, MO, United States). Synta66 was purchased from Glixx Laboratories (Southborough, MA, United States). YM-58483 and ML-9 hydrochloride were purchased from Tocris (Minneapolis, MN, United States). TG, 2-APB, synta66, YM-58483, menthol, and AITC were dissolved in DMSO; capsaicin was dissolved in ethanol as stock solutions. All of them were further diluted in the bath solution as working solutions with a final 0.1% DMSO or ethanol.

### Statistical Analysis

Data are expressed as original traces or as mean ± SEM. Treatment effects were statistically analyzed with a one-way analysis of variance (ANOVA). When ANOVA showed a significant difference, pairwise comparisons between means were performed by the *post hoc* Bonferroni method. Paired or two-sample Student’s *t*-tests were used when comparisons were restricted to two means. Error probabilities of *P* < 0.05 were considered statistically significant. The statistical software Origin 9.0 was used to perform all statistical analyses.

## Results

### The SOC Family Is Expressed in DRGs

**FIGURE 1:**
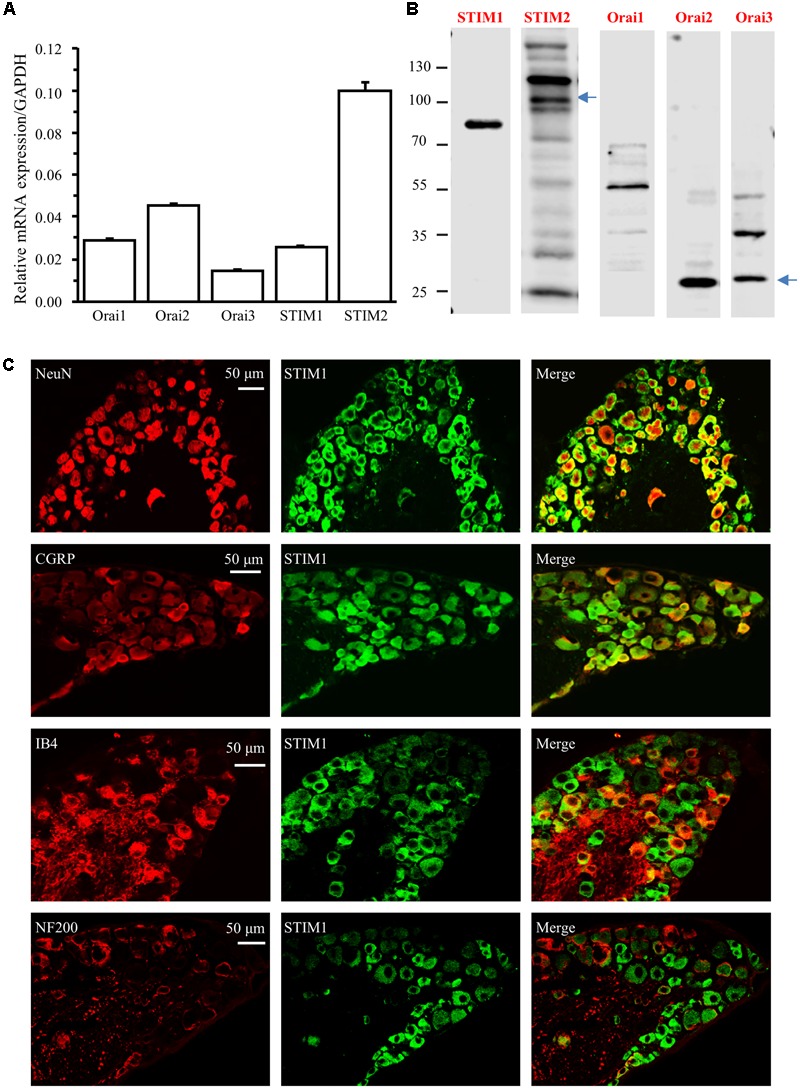
The SOC family is expressed in DRG. **(A)** Relative mRNA levels of Orai1, Orai2, Orai3, STIM1, and STIM2 in DRGs from adult mice. **(B)** Protein expression of STIM1, STIM2, Orai1, Orai2, and Orai3 in DRGs from adult mice. **(C)** Expression of STIM1 in different subtypes of DRG neurons. Co-localization of STIM1 (green) with NeuN (red), CGRP (red), IB4 (red), or NF200 (red) in DRG neurons. Images were captured under 30× magnification using a confocal microscope.

We and others have shown that YM-58483, a SOC inhibitor, attenuates acute, and chronic pain (Gao et al., 2013; Qi et al., 2016). Our previous studies have also demonstrated that SOCs are expressed and functional in spinal cord dorsal horn neurons and astrocytes (Xia et al., 2014; Gao et al., 2016). To better understand how SOCs are involved in the pain process, we examined the SOC family expression in DRGs. We first performed Taqman RT-qPCR in DRGs from adult mice and found that all mRNAs of the SOC family were expressed in DRGs (**Figure 1A**). To determine the protein expression of SOCs in DRGs, we performed Western blot analysis in DRGs. All SOC proteins were present in DRGs from adult mice (**Figure 1B**). These results suggest that the SOC family is expressed in DRGs.

To identify the specific types of DRG neurons expressing SOCs, we performed immunostaining experiments with DRGs collected from 4% paraformaldehyde-perfused adult mice. We took advantage of our previous identified the specific STIM1 antibody, and co-stained DRG sections with antibodies against STIM1 and NeuN (a neuron-specific marker). STIM1 staining was observed in the majority of DRG neurons (**Figure 1C**). To distinguish STIM1 expression between A- and C-fiber neurons, we co-stained STIM1 with neurochemical markers NF200 (for A fiber neurons), CGRP and IB4 (for C fiber neurons) (Ruscheweyh et al., 2007; Kestell et al., 2015). All CGRP positive neurons were STIM1 positive while the majority of IB4 positive neurons were co-stained with STIM1 (**Figure 1C**). Interestingly, STIM1 expression was only observed in 33.5% of NF200 positive neurons (**Figure 1C**). These results indicate that STIM1 is mainly expressed in C-fiber DRG neurons and, to a lesser extent, in A-fiber DRG neurons.

### Functional Characterization of SOCs in Different Types of DRG Neurons

**FIGURE 2:**
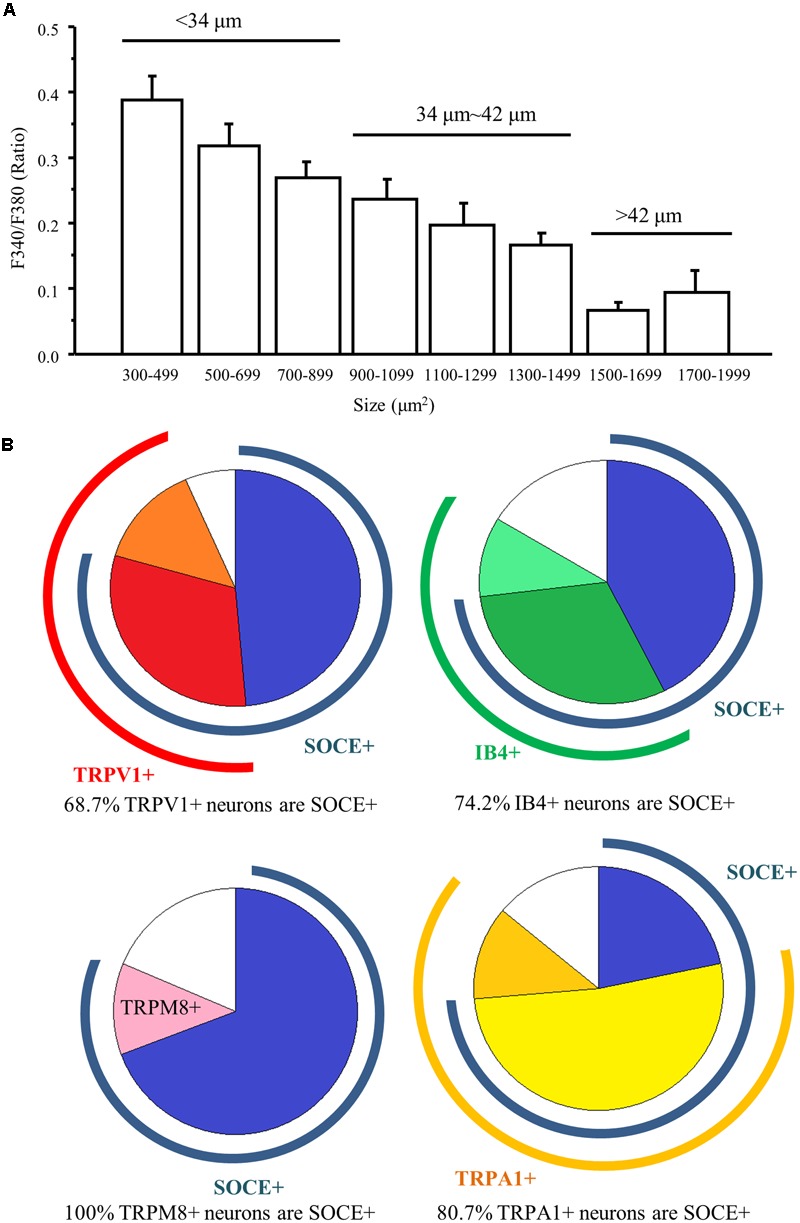
The function of SOCs in different types of DRG neurons. **(A)** Summary of SOCE in DRG neurons of different sizes. **(B)** Pie charts of SOCE positive DRG neurons overlapping with different types DRG neurons.

To determine the functional characterization of SOCs in DRG neurons, we performed Ca^2+^ imaging recordings and compared SOCE among different-diameter DRG neurons. Neurons were perfused with the Ca^2+^ free choline-Cl-based bath solution for 2 min, which was followed by 2 μM TG perfusion to induce intracellular Ca^2+^ store depletion. After TG treatment for 4 min, neurons were exposed to 2 mM Ca^2+^ choline-Cl-based solution to induce SOCE. SOCE was negatively correlated with the soma size of DRG neurons. SOCE was more robust when the size of the neurons was smaller (**Figure 2A**). Since nociceptors are often associated with small diameter neurons, we further defined the characteristics of SOCE positive neurons. Using nociceptor activators capsaicin (a TRPV1 agonist) (300 nM), menthol (a TRPM8 agonist) (100 μM), and AITC (a TRPA1 agonist) (100 μM) (Bandell et al., 2004; McKemy, 2007; Li et al., 2015), we found that 90 out of 131 capsaicin responding neurons had SOCE. In the total number of 316 tested neurons, only 38 neurons responded to menthol, but all menthol responding neurons had SOCE. Sixty-seven (67) out of 83 AITC responding neurons were SOCE positive. To confirm the co-immunostaining result of IB4 with STIM1, we examined IB4 staining after SOCE recordings and observed that 138 out of 186 IB4 positive DRG neurons had SOCE. These results suggest that SOCs are functional in a majority of nociceptors (**Figure 2B**).

### Depletion of ER Ca^2+^ Stores Results in STIM1 and STIM2 Translocation

**FIGURE 3:**
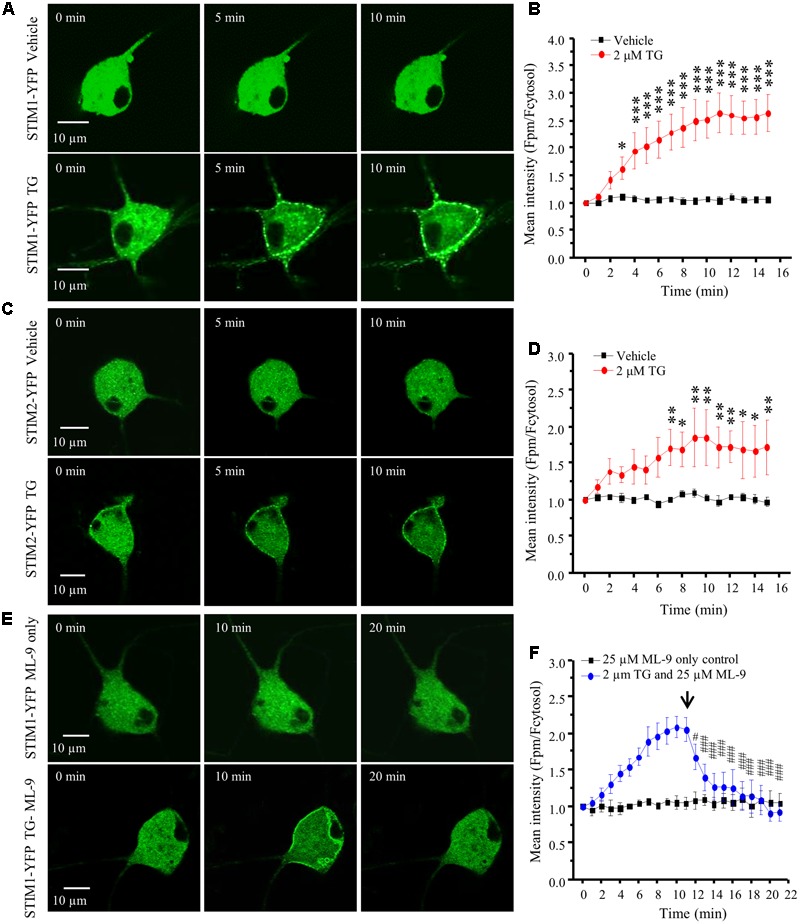
TG induced STIM1/2 translocation in DRG neurons transfected with STIM1- or STIM2-YFP. **(A)** Representative images of STIM1-YFP positive DRG neurons treated with vehicle (0.1% DMSO) or 2 μM TG at different time points. **(B)** Summary results of STIM1 translocation in neurons treated with vehicle and TG. **(C)** Representative images of STIM2-YFP positive DRG neurons treated with vehicle or 2 μM TG at different time points. **(D)** Summary results of STIM2 translocation in neurons treated with vehicle and TG. **(E)** Representative images of STIM1-YFP positive DRG neurons treated with 25 μM ML-9 only or 2 μM TG and then with 25 μM ML-9 at different time points. **(F)** Summary results of STIM1 translocation in neurons treated with TG and then ML-9. The fluorescence intensity was measured by ImageJ software and two lines were drawn manually in the cytosol and plasma membrane (PM), respectively. The ratio of the PM/cytosolic pixel intensities was calculated. Values represent mean ± SEM; *n* = 6–8 neurons; ^∗^*P* < 0.05, ^∗∗^*P* < 0.01, ^∗∗∗^*P* < 0.001 compared with 0 min point in **(B,D)**; ^#^*P* < 0.05, ^###^*P* < 0.001 compared with 11 min point in **(F)** by One-way ANOVA.

STIM1 can be activated by intracellular Ca^2+^ store depletion in cell lines (Stathopulos et al., 2006; Berna-Erro et al., 2009). We have also demonstrated in our previous study that depletion of ER Ca^2+^ stores results in STIM1 puncta formation in spinal cord astrocytes (Gao et al., 2016). To determine whether this phenomenon also occurs in DRG neurons, we transfected acutely dissociated DRG neurons with STIM1-YFP, cultured for 24–48 h and performed live cell confocal imaging. Transfection of STIM1-YFP into DRG neurons led to STIM1-YFP expression throughout the cell body and axonal branches except the nucleus. Application of vehicle did not induce STIM1 movement, while 2 μM TG induced robust STIM1 translocation from cytosol toward the plasma membrane in a time-dependent manner (**Figures 3A,B**). The significant effect was observed 3 min after TG treatment. Similarly, we transfected DRG neurons with STIM2-YFP, and STIM2 translocation was also observed after TG treatment (**Figures 3C,D**), but to a lesser extent. Our results demonstrated that both STIM1 and STIM2 can be activated in response to the depletion of intracellular Ca^2+^ stores. As ML-9 is a potent SOC inhibitor, and more specific for STIM1 (Smyth et al., 2008), we applied 25 μM ML-9 to the STIM1-YFP transfected neurons. ML-9 alone did not affect STIM1 puncta formation, but completely reversed TG-induced STIM1 puncta formation within 6 min (**Figures 3E,F**).

### STIM1, STIM2, Orai1, and Orai3 Are Responsible for SOCE in DRG Neurons

**FIGURE 4:**
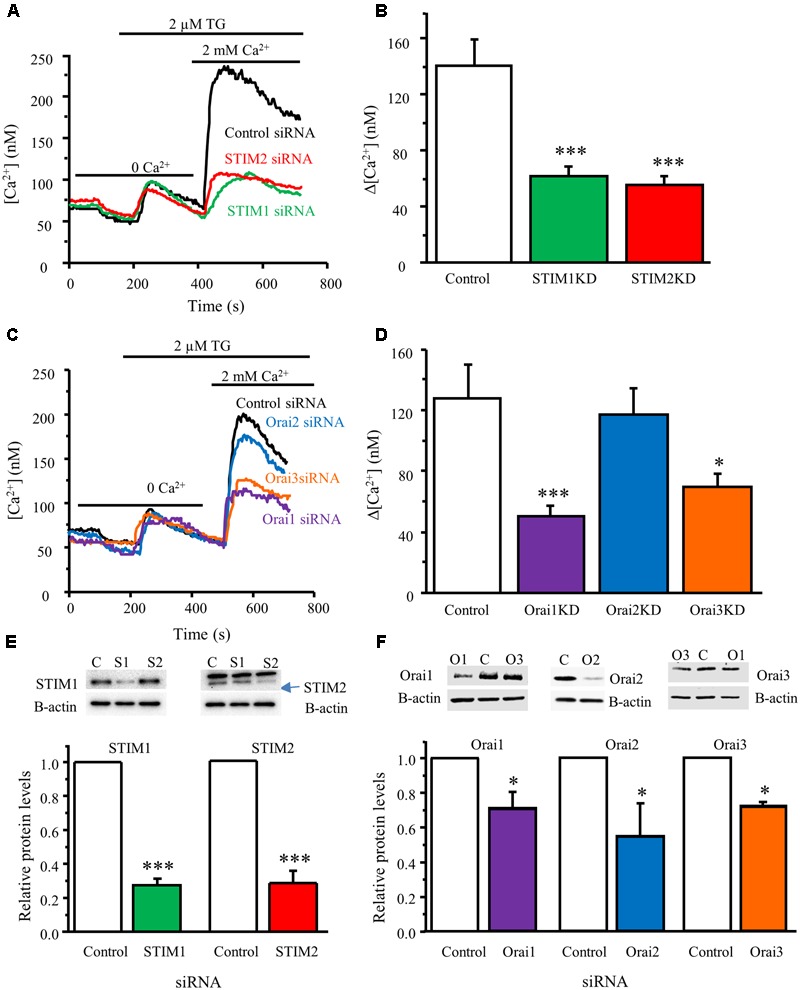
STIM1, STIM2, Orai1, and Orai3 contribute to SOCE in DRG neurons. **(A)** Representative traces of SOCE in DRG neurons treated with control siRNA, STIM1 siRNA or STIM2 siRNA. **(B)** Summary result from **(A)**, control group *n* = 15 neurons, STIM1KD group *n* = 18, STIM2KD group *n* = 13. **(C)** Representative traces of SOCE in neurons treated with control siRNA, Orai1 siRNA, Orai2 siRNA, Orai3 siRNA. **(D)** Summary of effects of control siRNA (*n* = 21), Orai1 siRNA (*n* = 29), Orai2 siRNA (*n* = 29), Orai3 siRNA (*n* = 28) on SOCE in DRG neurons. **(E)** Western blot analysis confirming that STIM1 and STIM2 were significantly knocked down by their specific siRNAs. **(F)** Western blot analysis confirming that Orai1, Orai2, and Orai3 were significantly knocked down by their specific siRNAs. Values represent mean ± SEM; ^∗^*P* < 0.05, ^∗∗∗^*P* < 0.001 compared with control by One-way ANOVA in **(B,D)** and by Student’s *t*-test in **(E,F)**.

To determine whether activated STIM proteins mediate SOCE in DRG neurons, we used the RNA interference gene silencing approach. Neurons from neonatal mice were co-transfected with GFP plasmid and STIM1, STIM2, or control siRNA. Western blot analysis was conducted 48 hours after transfection to validate knockdown efficiency. STIM1 siRNA dramatically reduced STIM1 protein level (**Figure 4E**), and had no effect on STIM2 expression. Similarly, STIM2 siRNA drastically decreased STIM2 protein level (**Figure 4E**), and had no effect on STIM1 expression. Ca^2+^ imaging was performed in GFP-positive neurons 72 h after transfection. TG-induced SOCE was significantly decreased by knockdown of STIM1 or STIM2 (**Figures 4A,B**), while TG-induced Ca^2+^ release was not affected (data not shown). These results suggest that both STIM1 and STIM2 contribute to SOCE in DRG neurons.

To identify which pore-forming subunits of SOCs are involved in SOCE in DRG neurons, we knocked down Orai1, Orai2, or Orai3 using their specific siRNAs targeting Orai1, Orai2, or Orai3. Knockdown of Orai1 or Orai3 significantly decreased SOCE in neonatal DRG neurons (SOCE was reduced by 60 or 45%, respectively), while knockdown of Orai2 had no such effect (**Figures 4C,D**). We also confirmed that Orai1, Orai2, and Orai3 protein expression levels were significantly reduced in DRG neurons transfected with their respective siRNAs that did not interfere with each other (**Figure 4F**). These results demonstrate that Orai1 and Orai3 are responsible for SOCE in DRG neurons.

Based on this finding, we hypothesized that STIM1, STIM2, Orai1 and Orai3 mediate SOCE in adult DRG neurons. To test this hypothesis, we transfected specific siRNAs targeting STIM1, STIM2, Orai1 or Orai3, respectively, into adult mouse DRG neurons. Similar to what we observed in neonatal neurons, SOCE was significantly decreased in those knockdown neurons (identified by GFP expression) (**Supplementary Figure S1**), and the degree of decrease in SOCE was also similar to that observed in neonatal neurons. This result supports our hypothesis that STIM1, STIM2, Orai1, and Orai3 also contribute to SOCE in adult DRG neurons.

### Pharmacological Properties of TG-Induced Ca^2+^ Entry in DRG Neurons

**FIGURE 5:**
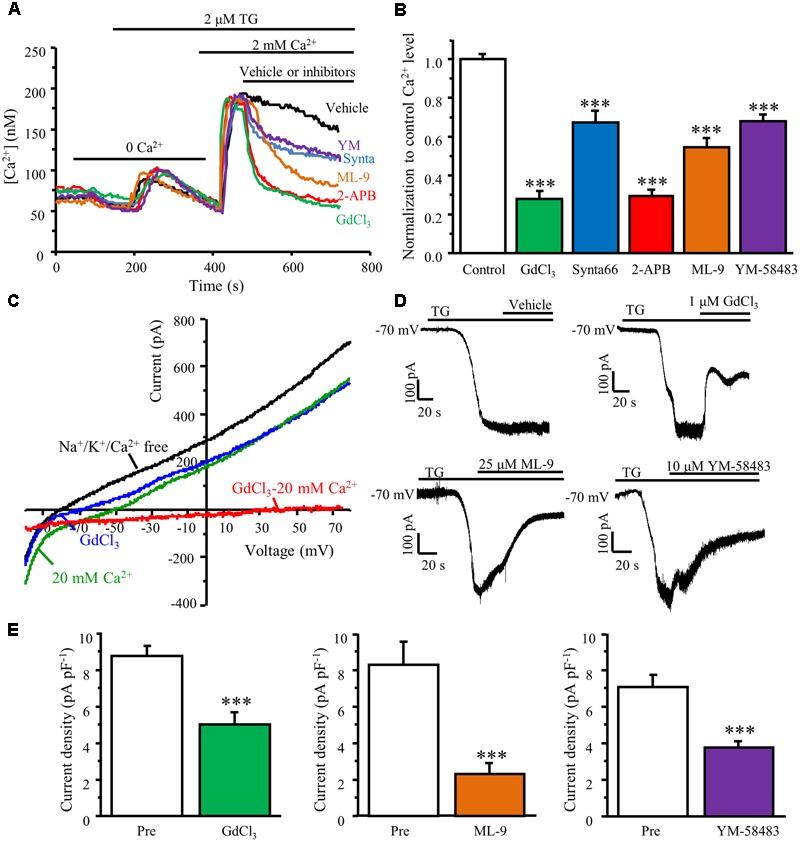
TG-induced Ca^2+^ entry is attenuated by SOC inhibitors. **(A)** Representative traces of SOCE in DRG neurons treated with SOC inhibitors. **(B)** Summary of effects of SOC inhibitors on SOCE in DRG neurons, *n* = 18–41. **(C)** Representative I–V relationships of TG-induced SOC currents. The currents were recorded in Na^+^/K^+^/Ca^2+^ free solution (black), 20 mM Ca^2+^ solution (green), and after application of GdCl_3_ (blue). An inward Ca^2+^-dependent current was obtained by subtracting the current recorded after the application of GdCl_3_ from before this application (red). **(D)** Representative TG-induced SOC currents recorded in the presence of vehicle, GdCl_3_, ML-9 or YM-58483. **(E)** Summary of inhibition of TG–induced SOC currents by GdCl_3_ (*n* = 11), ML-9 (*n* = 10), or YM-58483 (*n* = 12). Values represent mean ± SEM; ^∗∗∗^*P* < 0.001 compared with control by One-way ANOVA in **(B)** and by Student’s *t*-test in **(E)**.

We and others have reported that Orai1 is the only pore forming subunit responsible for SOCE in dorsal horn neurons and other native cell types (Braun et al., 2009; Maul-Pavicic et al., 2011; Xia et al., 2014). To characterize the pharmacological properties of Orai1/Orai3-mediated SOCE in DRG neurons, we examined the effects of YM-58483, 2-APB, GdCl_3_, synta66 (CRAC channel inhibitors), and ML-9 (STIM1 inhibitor) on TG-induced Ca^2+^ response in DRG neurons. TG-induced SOCE was recorded in cultured DRG neurons using our Ca^2+^ imaging system. Once SOCE reached maximal level, 25 μM ML-9, 30 μM synta66, 30 μM 2-APB, 10 μM YM-58483, or 1 μM GdCl_3_ were applied immediately. YM-58483, synta66, and ML-9 significantly attenuated TG-induced Ca^2+^ entry in DRG neurons while both 2-APB (30 μM) and GdCl_3_ drastically reduced SOCE in DRG neurons (**Figures 5A,B**), suggesting that SOCs in DRG neurons are endowed with pharmacological properties similar to dorsal horn neurons and other non-neuronal cells.

To confirm these Ca^2+^ imaging results, whole-cell patch clamp recordings were also performed. SOC currents were generated by a voltage ramp protocol. Neurons were held at 0 mV to inactivate voltage-gated Ca^2+^ channels. Currents were recorded in a NMDG-based Na^+^/K^+^/Ca^2+^ free solution after whole-cell recording configuration was established in Tyrode’s solution. Voltage-activated currents were recorded in the absence of Na^+^/K^+^/Ca^2+^ for at least 5 min to allow BAPTA and TG diffusion from the pipette solution. Bath application of 20 mM Ca^2+^ increased inward currents and decreased outward currents, which was partially blocked by 1 μM GdCl_3_. When subtracting currents recorded in GdCl_3_ from those obtained in the 20 mM Ca^2+^ solution, TG-induced small SOC currents with a reversal potential around 60 mV was observed (**Figure 5C**). To better evaluate the effects of SOC inhibitors on SOC currents, we used a divalent-free solution since SOC currents are relatively larger in the divalent-free solution due to the permeability of the cell membrane to K^+^ and Na^+^ (DeHaven et al., 2007; Gemes et al., 2011). The DRG neurons were held at -70 mV and a gap free recording protocol was used. SOC currents were induced by intracellular application of BAPTA/TG and recorded in the divalent free solution for at least 5 min. Once the I_CRAC_ reached maximal levels, 1 μM GdCl_3_, 25 μM ML-9, or 10 μM YM-58483 were applied. Inhibition of SOCs significantly diminished BAPTA/TG induced currents in DRG neurons (**Figures 5D,E**). These data reaffirmed the Ca^2+^ imaging results.

### Orai1 and Orai3 May Form Homomultimers in DRG Neurons

**FIGURE 6:**
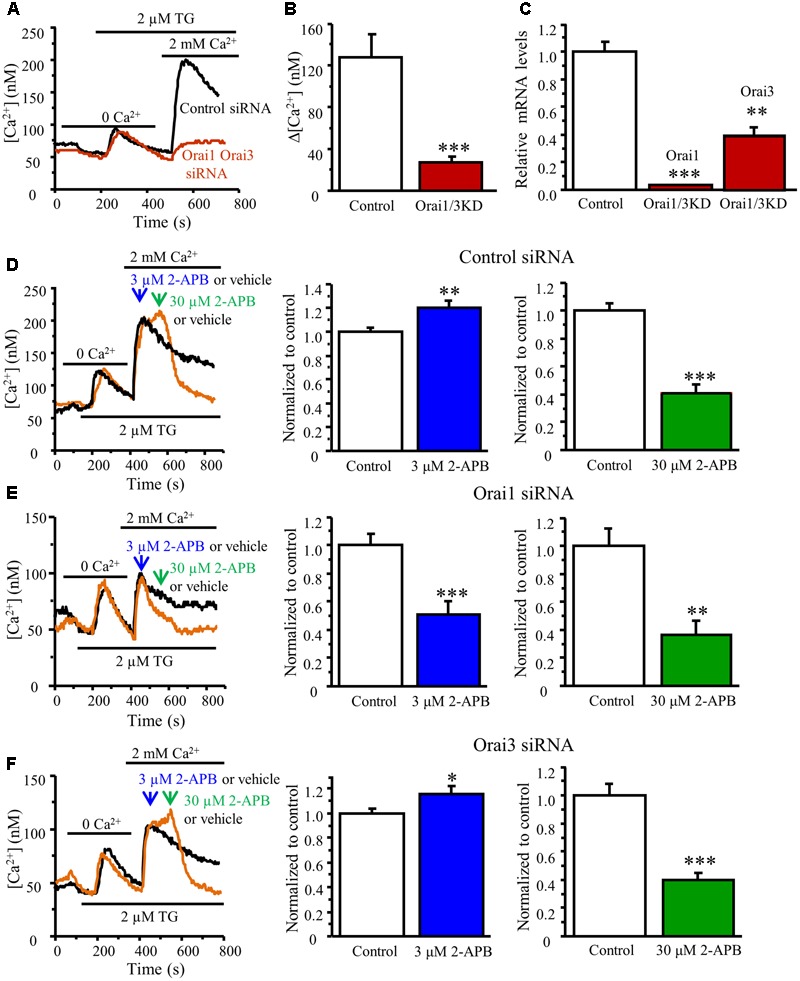
Effects of different concentrations of 2-APB on TG-induced Ca^2+^ entry in DRG neurons transfected with Orai1 and Orai3 siRNA. **(A)** Representative traces of SOCE in neurons treated with control siRNA and Orai1 plus Orai3 siRNAs. **(B)** Summary of effects of control siRNA (*n* = 21) and Orai1 plus Orai3 siRNAs (*n* = 12) on SOCE in DRG neurons. **(C)** RT-qPCR confirming that both Orai1 and Orai3 were significantly knocked down by transfection with Orai1 plus Orai3 siRNAs. **(D–F)** Effects of vehicle (control), 3 and 30 μM 2-APB on SOCE in DRG neurons transfected with control siRNA **(D)**, Orai1 siRNA **(E)** Orai3 siRNA **(F)**, *n* = 14–29. Values represent mean ± SEM; ^∗^*P* < 0.05, ^∗∗^*P* < 0.01, ^∗∗∗^*P* < 0.001 compared with control group by Student’s *t*-test.

Since both Orai1 and Orai3 contribute to SOCE in DRG neurons, it raises a question of whether Orai1 and Orai3 form homomultimers or heteromultimers in DRG neurons. To answer this question, we performed a double knockdown of Orai1 and Orai3. Knockdown of both Orai1 and Orai3 drastically attenuated SOCE in DRG neurons (SOCE was reduced by 80%) (**Figures 6A–C**), suggesting they may form homomultimers since the effect of both knockdown is greater than that of either Orai1 or Orai3 knockdown. To further confirm this result, we used a low concentration of 2-APB, which enhances Orai1-mediated SOCE (Navarro-Borelly et al., 2008; Ali et al., 2017). As expected, bath application of 3 μM 2-APB significantly enhanced SOCE in control siRNA transfected DRG neurons (**Figure 6D**), and this increase was abolished when Orai1 was knocked down (**Figure 6E**). However, 2-APB-induced increase in SOCE was still observed in Orai3 knockdown neurons (**Figure 6F**), indicating that Orai1 and Orai3 likely form homomultimers to mediate SOCE in DRG neurons.

### Activation of SOCs by TG Increases Neuronal Excitability in DRG Neurons

**FIGURE 7:**
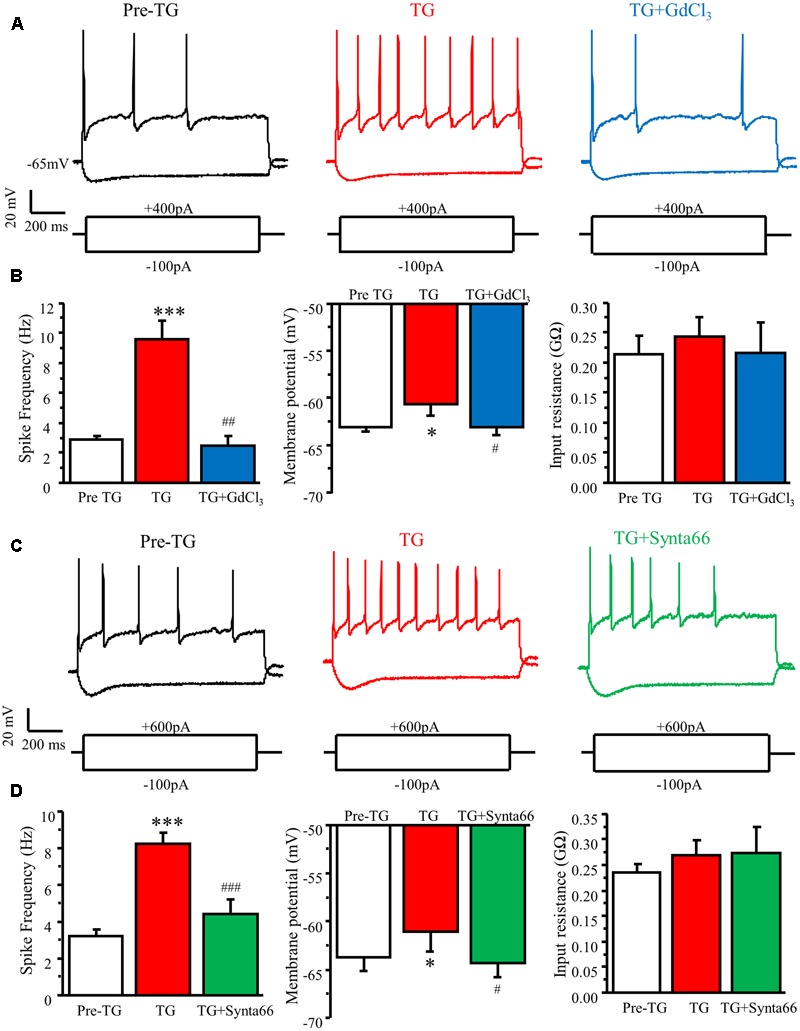
Activation of SOCs by TG increases neuronal excitability in DRG neurons. **(A)** Representative action potentials recorded in DRG neurons before (Pre TG), 5 min after TG application (TG), and 3 min after GdCl_3_ addition (TG+GdCl_3_). **(B)** Summary of TG-induced changes in spike frequency, membrane potential, and input resistance and attenuation by GdCl_3_ in DRG neurons. **(C)** Representative action potentials recorded in DRG neurons before (Pre TG), 5 min after TG application (TG), and 3 min after synta66 addition (TG+synta66). **(D)** Summary of TG-induced changes in spike frequency, membrane potential, and input resistance and attenuation by synta66 in DRG neurons. Values represent mean ± SEM; *n* = 6–12 neurons; ^∗^*P* < 0.05, ^∗∗∗^*P* < 0.001 compared with Pre TG group, ^#^*P* < 0.05, ^##^*P* < 0.01, ^###^*P* < 0.001 compared with TG group by Student’s *t*-test.

To explore the functional significance of SOC activation in DRG neurons, we performed current-clamp recordings in cultured adult mouse DRG neurons. Action potentials were induced by current injections from a holding potential of -65 mV. To assess the effects of TG on neuronal excitability in DRGs, neurons were maintained at the same holding potential of -65 mV, and a certain amplitude of current was injected by which 2 to 5 action potentials could be evoked. TG significantly increased spike frequency, caused membrane depolarization, but had no effect on steady-state input resistance (**Figure 7**). To confirm that the modulation of action potential induced by TG is mediated by SOCE, 1 μM GdCl_3_, or 30 μM synta66 were applied to the bath 5 min after TG addition. We found that both GdCl_3_ and synta66 reversed TG’s effect on spike frequency and membrane depolarization (**Figure 7**). These results indicate that TG-induced activation of SOCE increases neuronal excitability of DRG neurons.

### Orai1 and Orai3 Are Required for TG-Induced Modulation of Neuronal Excitability in DRG Neurons

**FIGURE 8:**
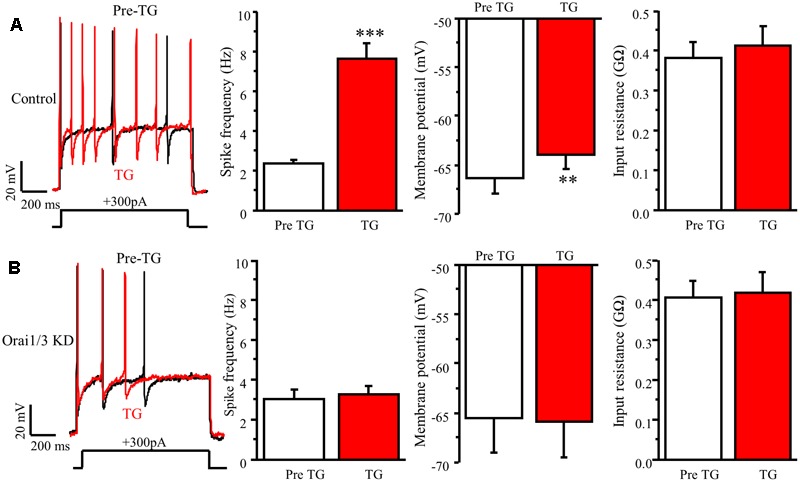
Orai1 and Orai3 are required for TG-induced modulation of neuronal excitability in DRG neurons. **(A)** TG-induced modulation of action potentials before (Pre TG) and 5 min after TG application in DRG neurons transfected with control siRNA, *n* = 8. **(B)** TG-induced modulation of action potentials before (Pre TG) and 5 min after TG application in DRG neurons transfected with Orai1 siRNA plus Oria3 siRNA, *n* = 8. Values represent mean ± SEM; ^∗∗^*P* < 0.01, ^∗∗∗^*P* < 0.001 compared with Pre TG group by Student’s *t*-test.

To further confirm whether Orai1 and Orai3 contribute to neuronal excitability in DRG neurons, we did double knockdown of Orai1 and Orai3 by transfecting both Oria1 siRNA and Orai3 siRNA with GFP into neonatal DRG neurons. Current-clamp recordings were performed in GFP positive neurons 72 h after transfection. TG-induced increase of spike frequency and membrane depolarization in control group were absent in the Orai1 and Orai3 double knockdown neurons (**Figure 8**). The double knockdown efficiency was confirmed by RT-qPCR (**Figure 6C**). These findings suggest that Orai1 and Orai3 are essential for SOCE-mediated neuronal excitability in DRG neurons.

## Discussion

Findings from this study have demonstrated that the SOC family is expressed in DRG and have revealed that STIM1, STIM2, Orai1, and Orai3 mediate SOCE in DRG neurons. We have also found that STIM1 is mainly expressed in nociceptors, and that SOCE is more robust in small- and medium-sized DRG neurons. Most importantly, activation of SOCs increases neuronal excitability, which may contribute to the pain process.

We have found that SOCs are mainly functional in nociceptors including TRPV1, TRPM8, and TRPA1 positive neurons. As TRPV1, TRPM8, and TRPA1 channels are all identified to be involved in thermal sensation and inflammatory pain, these results support our previous report that SOCs may be involved in pain (Gao et al., 2013).

As documented, depletion of ER Ca^2+^ stores can induce a significant intracellular redistribution of STIM1 from ER to discrete areas located underneath the plasma membrane to form puncta (Baba et al., 2006; Fahrner et al., 2013), which causes activation of the Orai channels at the plasma membrane mediating Ca^2+^ influx from extracellular milieu. Although DRG neurons are relatively difficult to transfect, we were able to transfect STIM1-YFP or STIM2-YFP to DRG neurons and performed live-cell confocal microscopy on these transfected neurons. Our data revealed that depletion of ER Ca^2+^ stores by TG induced a robust translocation of STIM1 from cytosol toward plasma membrane, which was reversed by the STIM1 inhibitor ML-9. We also observed TG-induced STIM2 translocation, but to a lesser extent, suggesting that both STIM1 and STIM2 can be activated upon ER Ca^2+^ store depletion.

It has been reported that STIM1 and Orai1 are the key components responsible for SOCE in many non-excitable cell types (Targos et al., 2005; McCarl et al., 2010; Putney et al., 2017); STIM1, STIM2, and Orai1 are the main subunits of SOCs mediating SOCE in CNS neurons (Berna-Erro et al., 2009; Klejman et al., 2009; Xia et al., 2014). Our RT-qPCR and Western blot results have demonstrated that the SOC family members are differentially expressed in DRGs from adult mice. The mRNA level of STIM2 is greater than that of STIM1, which is consistent with previous reports that STIM2 is the dominant isoform in the nervous system (Skibinska-Kijek et al., 2009; Xia et al., 2014). Surprisingly, our knockdown results show that both STIM1 and STIM2 are involved in SOCE in DRG neurons, and that they are equally necessary for SOCE. By way of contrast, STIM2 has no effect or an inhibitory effect on SOC activation in other cell types (Soboloff et al., 2006; Brechard et al., 2009). Whether STIM1 and STIM2 form heteromultimers mediating SOCE still need to be determined. While Orai1 is the only subunit mediating SOCE in most cell types (Hewavitharana et al., 2007; McCarl et al., 2009; Xia et al., 2014; Gao et al., 2016), our results reveal that not only Orai1 but Orai3 also contributes to SOCE in DRG neurons. As Orai1 and Orai3 have also been identified as ARC channels, where they form a heteropentameric complex to mediate store-independent Ca^2+^ entry (Shuttleworth, 1996; Mignen et al., 2009; Thompson and Shuttleworth, 2013), we further explored the possibility that Orai1 and Orai3 form heteromultimers in DRG neurons using the siRNA knockdown approach and a pharmacological tool (a low concentration of 2-APB). We observed 3 μM 2-APB induced potentiation of SOCE in control neurons, which was abolished in Orai1 knockdown DRG neurons, but not in Orai3 knockdown neurons. In addition, knockdown of both Orai1 and Orai3 further reduced SOCE compared with Orai1 or Orai3 knockdown alone. Together, these data suggest that Orai1 and Orai3 likely form homomultimers to mediate SOCE in DRG neurons. We also noticed that SOCE was not completely abolished in the Orai1/3 double knockdown neurons, which might be due to the efficiency of the knockdown approach. It is also possible that other channels play a minor role in SOCE in DRG neurons, since a recent study using an shRNA knockdown method has shown that TRPC3 is involved in Orai-independent SOCE in primary nociceptors of rats (Alkhani et al., 2014). Nevertheless, Orai1 and Orai3 are the key pore forming subunits of SOCs mediating SOCE in DRG neurons.

Since the key pore-forming subunits of SOCs in DRG neurons are different from those in CNS neurons, we wondered whether the pharmacological properties of SOCs in DRG neurons were comparable to those in central neurons. Several structurally different SOC inhibitors GdCl_3_, 2-APB, synta66, ML-9, and YM-58483 were tested. All of these inhibitors attenuated TG-induced SOCE in DRG neurons. Consistently, our electrophysiology results showed that SOC currents were significantly diminished by SOC inhibitors GdCl_3_, ML-9, and YM-58483. We have noticed that the inhibition of SOCE by GdCl_3_ was greater than its reduction of SOC currents. In contrast, inhibition of SOC currents by ML-9 was more pronounced than the respective decrease in SOCE. These differences are probably due to the two different methods and extracellular solutions used to evaluate their effects. Although the key pore forming subunits of SOCs in DRG neurons are different from CNS neurons and other non-excitatory cell types (Targos et al., 2005; Berna-Erro et al., 2009; McCarl et al., 2010; Xia et al., 2014), the pharmacological properties are similar.

Findings from our current-clamp recordings indicated that depletion of ER Ca^2+^ stores resulted in membrane depolarization and firing rate increase in DRG neurons, which were eliminated by inhibition of SOCs, suggesting that SOCs produce an excitatory action in DRG neurons. This result is consistent with our previous report showing that activation of SOCs increases neuronal excitability (Xia et al., 2014), and is also supported by a previous study revealing that TRIM (a SOC inhibitor) blocks SOCE-mediated action potential firing in hypothalamus neurosecretory cells (Tobin et al., 2006). In contrast, a previous study showed that inhibition of SOCs increased neuronal excitability in which they incubated the DRGs with TRIM or vehicle for 30 min (Gemes et al., 2011). TRIM is not a specific inhibitor for SOCs, it is also a blocker for NO synthase (Handy et al., 1996). It has been reported that TRIM has anticonvulsant effects (Matsumura et al., 2008), therefore TRIM treatment may change neuronal excitability due to SOCE unrelated effects. Under our recording conditions, we examined TG-induced acute changes in neuronal excitability in the same neurons and compared the differences between pre- and post-TG application. Therefore, it is difficult to make a comparison between the two studies due to the different approaches. In this study, we further confirmed our results by recording action potentials in Orai1 and Orai3 double knockdown DRG neurons. Increased neuronal excitability induced by SOC activation was completely abolished in the double knockdown neurons, suggesting that SOCs exert an excitatory effect in DRG neurons. We previously showed that inhibition of SOCs could produce peripheral analgesic effect (Gao et al., 2013), suggesting that SOCs is involved in peripheral pain process. Our current-clamp results fit well with the behavioral study.

In summary, our results have demonstrated that SOCs are functionally expressed in small- and medium-sized DRG neurons. We have identified for the first time both Orai1 and Orai3 as the key pore forming subunits mediating SOCE in DRG neurons, which contribute to neuronal excitability. Our findings provide a potential mechanism for the peripheral analgesic effect of the SOC inhibitor YM 58483. The current study sheds light on the mechanisms by which SOCs modulate pain hypersensitivity and may suggest new treatments for chronic pain.

## Author Contributions

HH conceived of the project and designed experiments. DW designed and performed experiments, analyzed data, and prepared figures. YM and JX designed and performed some experiments. DW and HH prepared the manuscript. All authors read, revised, and approved the final manuscript.

## Conflict of Interest Statement

The authors declare that the research was conducted in the absence of any commercial or financial relationships that could be construed as a potential conflict of interest.

## Supplementary Material

FIGURE S1 TG-induced SOCE in adult DRG neurons transfected with STIM1, STIM2, Orai1, or Orai3 siRNAs. **(A)** Representative traces of SOCE in DRG neurons treated with control, STIM1 or STIM2 siRNA. **(B)** Summary result of **(A)**, *n* = 11–15. **(C)** Representative traces of SOCE in DRG neurons treated with control, Orai1 or Orai3 siRNA. **(D)** Summary result of **(C)**, *n* = 16–22. Values represent mean ± SEM; ^∗∗^*P* < 0.01, ^∗∗∗^*P* < 0.001 compared with control by One-way ANOVA.

## Abbreviations

AITC: allyl isothiocyanate
2-APB: 2-aminoethyl diphenyl borate
ARC: arachidonate-regulated Ca^2+^
CFA: complete Freund’s adjuvant
CGRP: calcitonin gene related peptide
CNS: central nervous system
CRAC channels: Ca^2+^ release-activated Ca^2+^ channels
DMSO: dimethyl sulfoxide
DRG: dorsal root ganglia
ER: endoplasmic reticulum
GFP: green fluorescent protein
HBSS: Hank’s balanced salt solution
IB4: isolectin B4
NMDG: 85 *N*-methyl-D-glutamine
SOCs: store-operated calcium channels
SOCE: store-operated calcium entry
TG: thapsigargin
