# Supplementary figures and images for: Orai1 and Orai3 Mediate Store-Operated Calcium Entry Contributing to Neuronal Excitability in Dorsal Root Ganglion Neurons
Source: Front Cell Neurosci. 2017 Dec 19;11:400. doi: 10.3389/fncel.2017.00400 (PMC5742109; doi:10.3389/fncel.2017.00400)

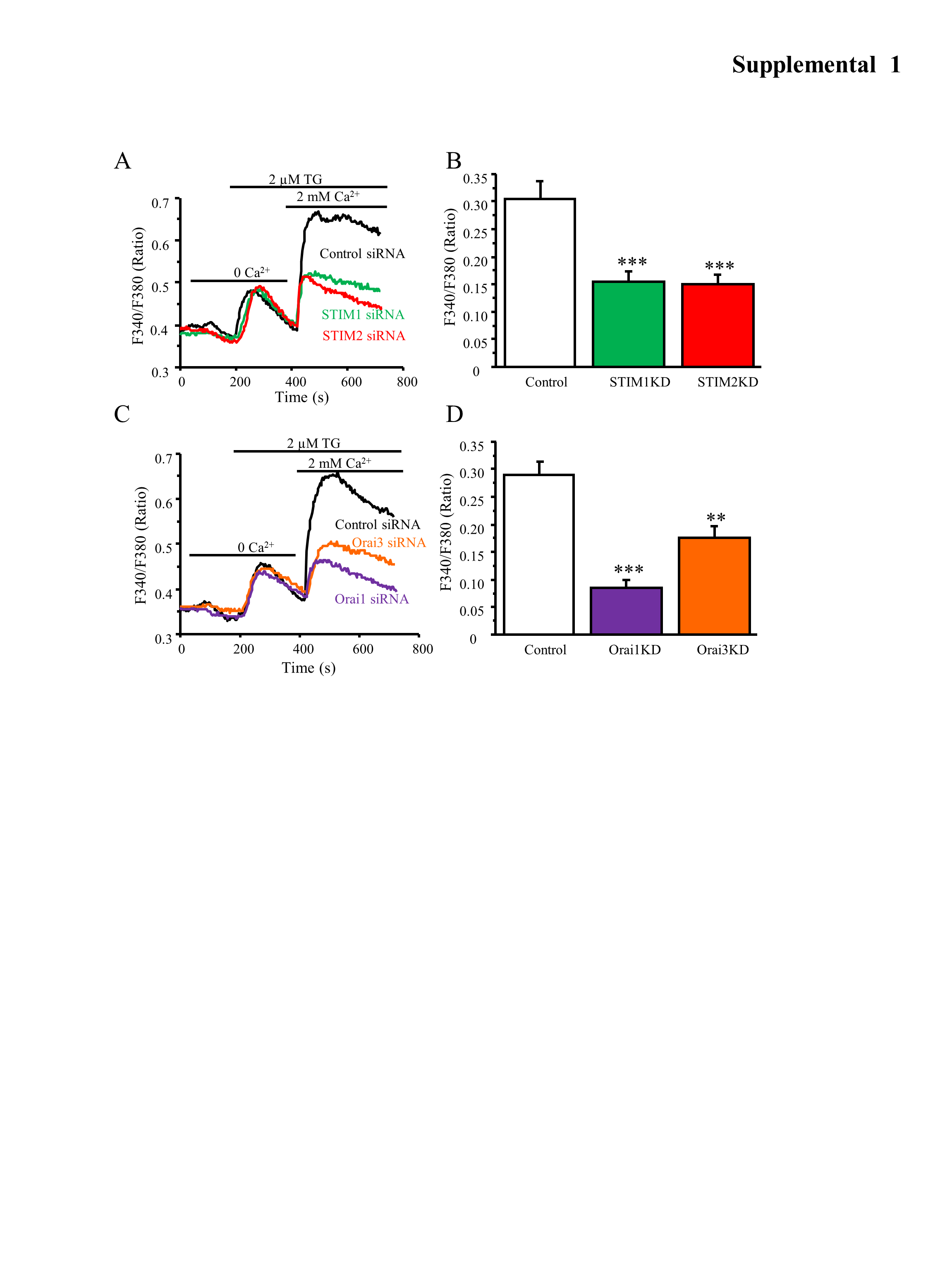

Supplement: FIGURE S1 — TG-induced SOCE in adult DRG neurons transfected with STIM1, STIM2, Orai1, or Orai3 siRNAs. (A) Representative traces of SOCE in DRG neurons treated with control, STIM1 or STIM2 siRNA. (B) Summary result of (A), n = 11–15. (C) Representative traces of SOCE in DRG neurons treated with control, Orai1 or Orai3 siRNA. (D) Summary result of (C), n = 16–22. Values represent mean ± SEM; ∗∗P < 0.01, ∗∗∗P < 0.001 compared with control by One-way ANOVA. [file Image_1.TIF]
